# PEP-1-CAT protects hypoxia/reoxygenation-induced cardiomyocyte apoptosis through multiple sigaling pathways

**DOI:** 10.1186/1479-5876-11-113

**Published:** 2013-05-06

**Authors:** Lei Zhang, Shuang Wei, Jun-Ming Tang, Ling-Yun Guo, Fei Zheng, Jian-Ye Yang, Xia Kong, Yong-Zhang Huang, Shi-You Chen, Jia-Ning Wang

**Affiliations:** 1 Institute of Clinical Medicine, Renmin Hospital, Hubei University of Medicine, Shiyan, Hubei 442000, China; 2 Department of Physiology and Key Lab of Human Embryonic Stem Cell of Hubei Province, Hubei University of Medicine, Hubei 442000, China; 3 Institute of Clinical Medicine and Department of Cardiology, Renmin Hospital, Hubei University of Medicine, Shiyan, Hubei 442000, China; 4 Department of Physiology & Pharmacology, University of Georgia, Athens, GA 30602, USA

**Keywords:** Cell-penetrating peptide, PEP-1, Catalase, Cardiomyocyte, Apoptosis, MAPK

## Abstract

**Background:**

Catalase (CAT) breaks down H_2_O_2_ into H_2_O and O_2_ to protects cells from oxidative damage. However, its translational potential is limited because exogenous CAT cannot enter living cells automatically. This study is aimed to investigate if PEP-1-CAT fusion protein can effectively protect cardiomyocytes from oxidative stress due to hypoxia/reoxygenation (H/R)-induced injury.

**Methods:**

H9c2 cardomyocytes were pretreated with catalase (CAT) or PEP-1-CAT fusion protein followed by culturing in a hypoxia and re-oxygenation condition. Cell apoptosis were measured by Annexin V and PI double staining and Flow cytometry. Intracellular superoxide anion level was determined, and mitochondrial membrane potential was measured. Expression of apoptosis-related proteins including Bcl-2, Bax, Caspase-3, PARP, p38 and phospho-p38 was analyzed by western blotting.

**Results:**

PEP-1-CAT protected H9c2 from H/R-induced morphological alteration and reduced the release of lactate dehydrogenase (LDH) and malondialdehyde content. Superoxide anion production was also decreased. In addition, PEP-1-CAT inhibited H9c2 apoptosis and blocked the expression of apoptosis stimulator Bax while increased the expression of Bcl-2, leading to an increased mitochondrial membrane potential. Mechanistically, PEP-1-CAT inhibited p38 MAPK while activating PI3K/Akt and Erk1/2 signaling pathways, resulting in blockade of Bcl2/Bax/mitochondrial apoptotic pathway.

**Conclusion:**

Our study has revealed a novel mechanism by which PEP-1-CAT protects cardiomyocyte from H/R-induced injury. PEP-1-CAT blocks Bcl2/Bax/mitochondrial apoptotic pathway by inhibiting p38 MAPK while activating PI3K/Akt and Erk1/2 signaling pathways.

## Introduction

Myocardial ischemia and reperfusion generate a large amount of reactive oxygen species (ROS) in cardiomyocytes subject to injury. ROS assaults intracellular organelles, cell membranes, and biological macromolecules including nucleic acid, protein, and lipid, resulting in oxidative stress and cell apoptosis [1,2]. Catalase (CAT) is one of essential enzymes metabolizing oxygen free radical via breakdown of H_2_O_2_ into H_2_O and O_2_, and thus protects cells from oxidative damage. However, exogenous CAT does not enter living cells automatically because of its poor permeability and cell membrane selectivity. Its translational value in protecting cells from oxidative stress damage, therefore, is very limited.

A great deal of efforts have been made to deliver full-length proteins into mammalian cells. Morris Group has designed a new type of PEP-1 peptide carrier (KETWWETWWTEWSQPKKKRKV) that enables the entering of large proteins into living cells [3]. In fact, several laboratories have successfully delivered full-length PEP-1 fusion proteins into cultured cells and nervous system by using this PEP-1 peptide carrier, including EGFP, β-Gal, antibodies, cyclophilin A, and human copper chaperone for Cu, Zn-SOD1 and CAT [4-7]. Our previous studies have demonstrated that PEP-1-CAT fusion proteins can be transduced into myocardium and protect against myocardial injury induced by ischemia-reperfusion in rats [8].

Cardiomyocyte apoptosis is an inevitable process during myocardial ischemia-reperfusion-induced injury [9]. We have previously reported an anti-apoptotic effect of PEP-1-CAT on H9c2 cardiomyocytes [10]. However, detailed mechanisms underlying the effect of PEP-1-CAT on H/R-induced H9c2 remain unknown. In the present study, we used the hypoxia-reoxygenation (H/R)-induced apoptosis model to investigate the mechanisms underlying the anti-apoptotic effect of PEP-1-CAT in H9c2 cells. H/R is a classic *in vitro* model mimicking myocardial ischemia-reperfusion injury *in vivo*. We found that PEP-1-CAT protected H9c2 from H/R-induced injury through blocking p38 MAPK activity and activating PI3K/Akt and Erk1/2 activity, which resulted in a blockade of Bax/Bcl-2/mitochondria apoptotic pathway and thus a reduction of cardiomyocyte apoptosis.

## Materials and methods

### Generation of biologically active PEP-1-CAT fusion protein

PEP-1-CAT fusion protein was isolated and purified as described by our laboratory previously [11]. Briefly, two prokaryotic expression plasmids for CAT and PEP-1-CAT were constructed using TA-cloning method. Both recombinant proteins were tagged with six histidine residues (His-tag) at the amino terminus. The two proteins were expressed and purified separately as described [11].

### Cell culture

H9c2 cells were cultured in Dulbecco’s modified Eagle’s medium(DMEM,Invitrogen) with 5 g/L glucose supplemented with 15% (v/v) fetal bovine serum (FBS, Hangzhou sijiqing Biological Engineering Materials Co., Ltd., China). Cells were routinely grown to subconfluency (>90% by visual estimate) in 75 cm^2^ flasks at 37°C in a humidified atmosphere with 5% CO_2_ prior to passage and seeding for experiments. To observe the morphological alteration, H9c2 cells were grown on cover slips and observed using a microscope (Nikon, Japan). To examine the aberrant nuclei in apoptotic cells, H9c2 cells were stained with 4,6-Diamidino-2-phenylinole (DAPI), and the nuclei were observed using a fluorescent microscope.

### Immunocytochemistry staining

H9c2 cells were grown to confluence in a 24-well plate and treated with purified PEP-1-CAT (2 μM) or CAT (2 μM). 6 h later, cells were washed twice with 1 × PBS and fixed with 4% paraformaldehyde for 15 min at room temperature. Immunocytochemistry staining was performed by using rabbit anti-Hisprobe (diluted 1:200) (Santa Cruz Biotechnology, USA) and mouse anti-Troponin T antibodies (diluted 1:200) (Santa Cruz Biotechnology, USA). Cells were then incubated with tetraethyl rhodamine isothiocyanate (TRITC)-conjugated rat anti-rabbit Ig G (diluted 1:250) and fluorescein isothiocyanate (FITC)-conjugated goat anti-mouse Ig G (diluted 1:250) at 25°C for 1 h. After washing for 3 times with PBS, cells were incubated with DAPI (Sigma, USA) for 10 min. The immunostained cells were observed with a fluorescent microscope (Nikon, Japan).

### Hypoxia-reoxygenation of H9c2 Cells

H9c2 cells were pretreated with or without PEP-1-CAT (2 μM) in low serum media (2% FBS) for 6 h followed by culturing in a low-oxygen condition (95% N_2_ + 5% CO_2_) for 21 h in a humidified hypoxia chamber (Stem Cell Technology, USA). After hypoxia incubation, the medium were replaced, and the cells were exposed to normal-oxygen condition (95% air + 5% CO_2_) for reoxygenation for 6 h [12]. Control cells were cultured in normoxic conditions. The supernatant and cells were collected separately for further analysis.

### Measurement of lactate dehydrogenase (LDH) and malondialdehy (MDA) levels

H9c2 cells were treated with PEP-1-CAT, harvested and lysed as previously described LDH release and MDA content were measured using commercial kits (JianCheng Bioengineering Institute, China).

### Superoxide anion production in H9c2

H9c2 cells were grown to confluence in a 24-well plate followed by H/R with CAT or PEP-1-CAT treatment. Cells were then split and cultured on cover slips and incubated with DHE (5 mM) (Beyotime Insitute of Brotechnology) at 37°C for 30 min. The DHE staining detecting superoxide anion production was observed using a fluorescent microscope (Nikon, Japan) or quantified by Flow Cytomety.

### Annexin V and PI binding assay

Annexin V and PI fluorescein staining kit (Bender MedSystems, Austria) were utilized to measure H9c2 cell apoptosis by following the manufacturer’s instruction. Briefly, 1 × 10^6^ cells were suspended in 200 μl 1 × binding buffer (10 mM HEPES pH 7.4, 140 mM NaCl, 2.5 mM CaCl_2_). Cells were then incubated with Annexin V (1:20) for 3 min followed by incubation with propidium iodide (PI, 1 mg/ml) for 15 min. Apoptosis rate was evaluated by Flow Cytometry.

### Measurement of mitochondrial membrane potential

Mitochondrial transmembrane potential was assessed using a sensitive fluorescent dye, a lipophilic cationic probe JC-1 (Invitrogen, USA). H9c2 cells were grown on cover slips and incubated with 5 mM JC-1 dye (Molecular Probes) at 37°C for 15 min. Cells were then washed with PBS for three times and analyzed immediately with a fluorescent microscope. Red emission indicates membrane potential-dependent JC-1 aggregates in mitochondria. Green fluorescence reflects the monomeric form of JC-1 appearing in cytoplasm after mitochondrial membrane depolarization.

### Quantitative reverse transcription polymerase chain reaction (qPCR)

Total RNA from H9c2 cells was extracted using TRIZOL Reagent (Invitrogen). RNA concentration was determined by UV spectrophotometry. qRT-PCR was performed using Thunderbird SYBR Master Mix (TOYOBO, Japan). Primer sequences were: Bcl-2: 5′-CGA CTT TGC AGA GAT GTC CA-3′ (forward), 5′-ATG CCG GTT CAG GTA CTC AG-3′ (reverse); Bax: 5′- CTG CAG AGG ATG ATT GCT GA-3′ (forward), 5′- GAT CAG CTC GGG CAC TTT AG-3′ (reverse); β-actin: 5′-GTC CAC CGC AAA TGC TTC TA-3′ (forward), 5′-TGC TGT CAC CTT CAC CGT TC-3′ (reverse). qPCR was performed on a Real-time PCR Detection System (Slan, Hongshi) with the following cycles: 95°C for 1 min, followed by 95°C for 15 s, 58°C for 15 s, and 72°C for 45 s for 40 cycles. β-actin expression was used as an internal control.

### Western blot analysis

Western blot was carried out to detect protein expression using following primary antibodies: rabbit anti-Bax (Santa Cruz Biotechnology), mouse anti-Bcl-2 (Santa Cruz Biotechnology), rabbit anti-Caspase-3 (Santa Cruz Biotechnology), rabbit anti-PARP-1 (Santa Cruz Biotechnology), rabbit anti-phospho-p38 MAPK (cell signaling technology), and rabbit anti-p38 MAPK (cell signaling technology). The protein expression levels were visualized using enhanced chemiluminescence method.

### Statistical analysis

All data are expressed as means ± SEM unless indicated otherwise. Differences among groups were determined by ANOVA. Differences between groups were determined by Student’s *t*-test with P < 0.05 considered statistically significant.

## Results

### PEP-1-CAT restored H/R-altered H9c2 cell morphology and decreased LDH and MDA levels

**Figure 1:**
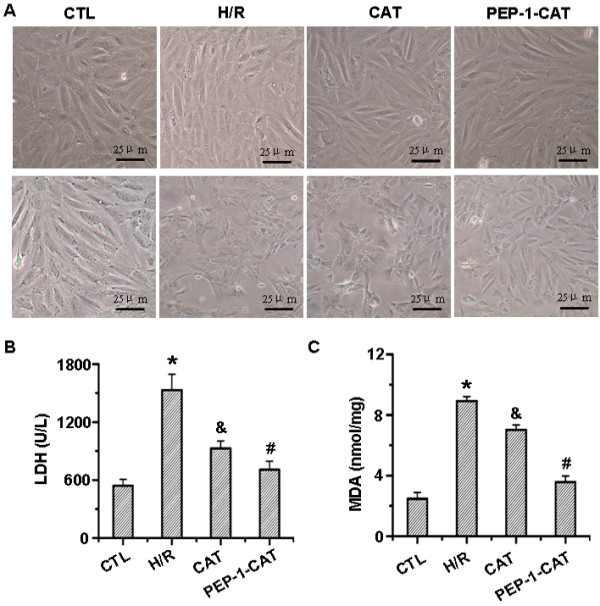
**PEP-1-CAT restored H/R-induced alteration of H9C2 cell morphology while inhibiting H/R-induced LDH release and MDA production.** (**A**) Effect of PEP-1-CAT on H9c2 cell morphology. Upper panel: cell morphology prior to H/R treatment; lower panel: cell morphology with H/R treatment. (**B**) PEP-1-CAT reduced LDH levels *P<0.01 vs control (CTL); ^&^P<0.05 vs H/R; ^#^P<0.01 vs H/R or H/R + CAT (CAT); n = 6. (**C**) PEP-1-CAT decreased MDA levels. *P<0.01 vs control (CTL);^&^P<0.05 vs H/R; ^#^P<0.01 vs H/R or H/R + CAT (CAT); n = 6.

PEP-1–CAT fusion proteins were successfully transduced into H9c2 cells as shown by immunocytochemistry staining (Additional file 1: Figure S1). CAT fusion proteins, however, were unable to be transduced into the cells (Additional file 1: Figure S1). H/R altered H9c2 cell morphology. H/R treatment changed the spindle-shaped and well-organized morphology to a shrink, round and distorted morphology. PEP-1-CAT transduction, however, almost restored the spindle-shaped morphology seen in the untreated cells (Figure 1A).

LDH release is an indicator of cellular injury. Compared to untreated cells, LDH levels were markedly increased by H/R injury. CAT transduction decreased LDH release. PEP-1-CAT transduction, however, had a greater impact on LDH levels compared to the CAT transduction (Figure 1B). MDA reflects cardiomyocyte oxidative damage. H/R treatment strikingly increased the MDA level, but PEP-1-CAT significantly decreased the MDA level (Figure 1C).

### PEP-1-CAT had a greater effect on superoxide anion production than CAT

**Figure 2:**
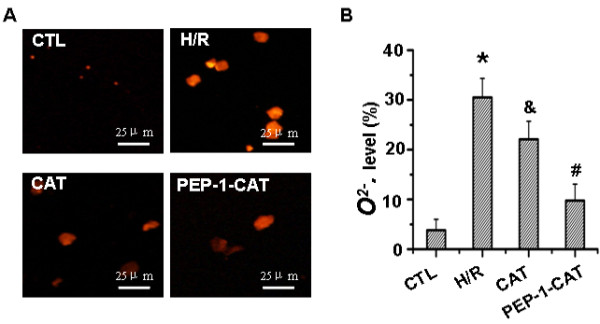
**PEP-1-CAT inhibited H/R-induced superoxide anion production.** (**A**) Superoxide anion production was observed using a fluorescent microscope. (**B**) Superoxide anion production was quantified by Flow Cytometry (n = 6). ^*^P<0.01 vs control (CTL); ^&^P<0.05 vs H/R; ^#^P<0.05 vs H/R or H/R + CAT (CAT); n = 4.

H/R treatment significantly increased superoxide anion production in H9c2 cells compared to the untreated group. CAT transduction slightly reduced superoxide anion level. PEP-1-CAT transduction, however, significantly inhibited the level of superoxide anion. These results demonstrated that PEP-1-CAT had a much stronger effect than CAT on removing superoxide anion from the injured cells (Figure 2).

### PEP-1-CAT attenuated H/R-induced H9c2 cell apoptosis

**Figure 3:**
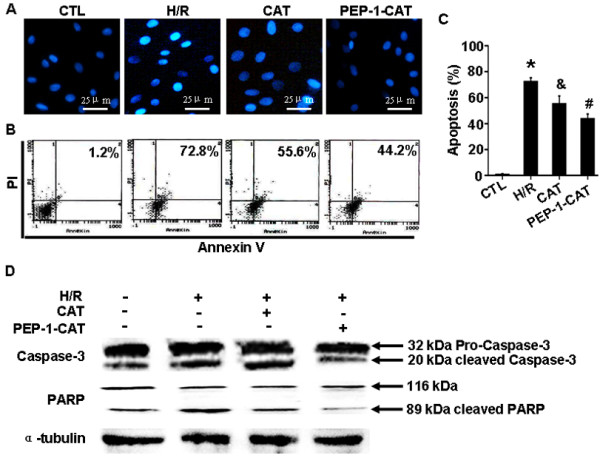
**PEP-1-CAT inhibited H/R-induced H9c2 cell apoptosis.** H9c2 cells were pretreated with CAT or PEP-1-CAT for 6 h and placed into normoxia environment for 27 h or into hypoxia chamber for 21 h followed by 6 h reoxygenation. (**A**) Cell apoptosis was detected by DAPI staining. (**B**-**C**) H/R-induced H9c2 apoptosis rate was quantiied by Flow Cytometry. *P<0.01 vs control (CTL);^&^P<0.05 vs H/R; ^#^P<0.01 vs H/R or H/R + CAT (CAT); n = 5. (**D**) PARP and caspase-3 protein expression was detected by western blot.

Comparing to the control group, significantly more cells underwent apoptosis as shown by the bright DAPI staining in H/R group. H/R treatment condensed the nuclei of H9c2 cells, an indicator of apoptosis. PEP-1-CAT transduction, however, restored H9c2 nuclei to the normal morphology (Figure 3A). Quantitative analysis using Flow Cytometry confirmed that PEP-1-CAT significantly inhibited H/R-induced apoptosis (Figure 3B-C). PolyADP-ribose polymerase-1 (PARP-1) is known to be involved in DNA damage while caspase-3 is known to regulate cell apoptosis. To determine whether PEP-1-CAT affects H/R-induced PARP and caspase-3 cleavage, we treated cells with H/R in the presence and absence of PEP-1-CAT and analyzed their cleavages using anti-PARP-1 and Caspase-3 antibodies. As shown in Figure 3D, H/R induced PARP and caspase-3 cleavage in H9c2 cells but the effects were inhibited by PEP-1-CAT, further demonstrating that PEP-1-CAT suppressed H/R-induced apoptosis.

### PEP-1-CAT regulated the expression of apoptosis-related proteins

**Figure 4:**
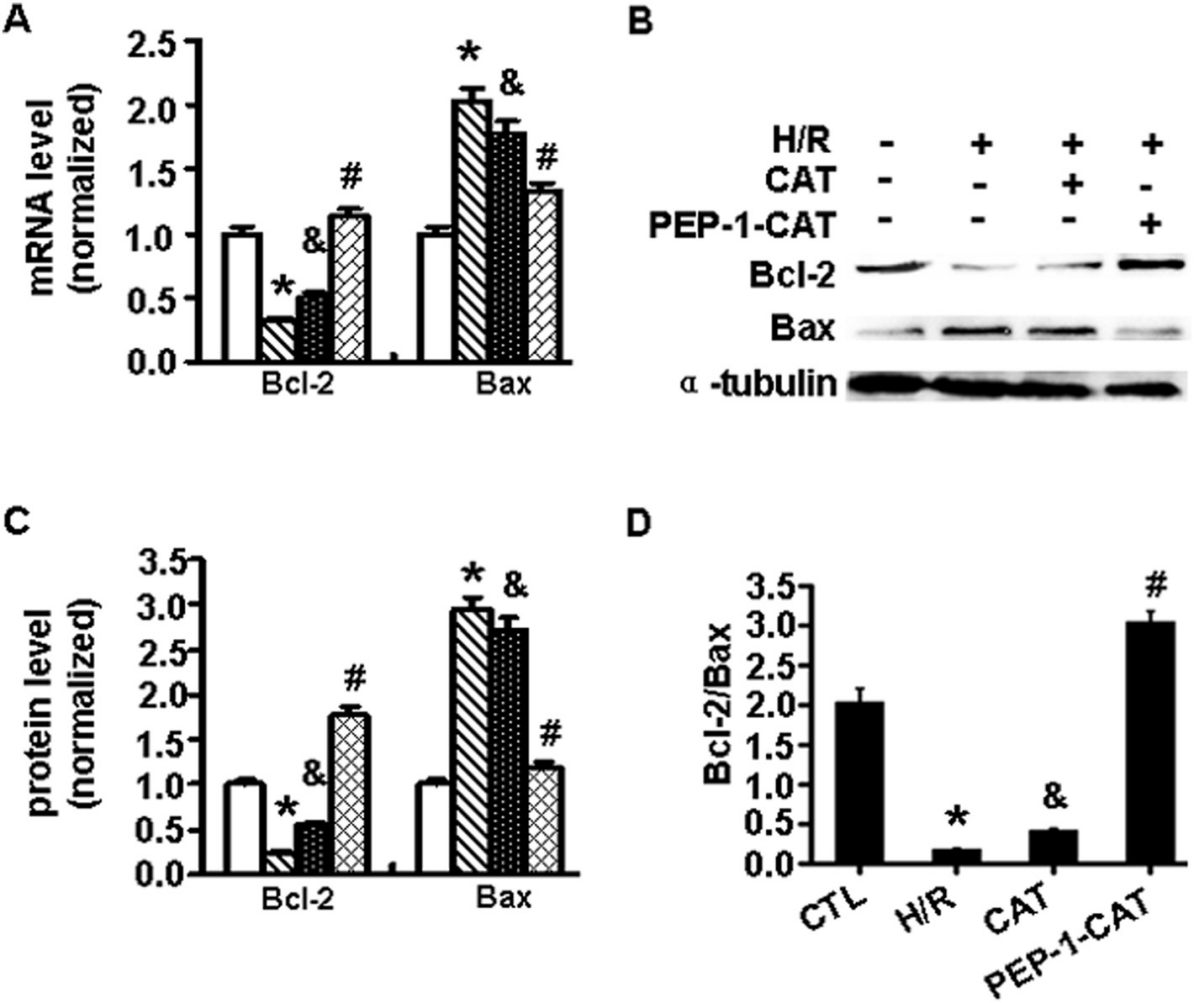
**PEP-1-CAT regulated Bcl-2 and Bax expression.** (**A**) Bcl2 and Bax mRNA expression was detected by qRT-PCR and normalized to β-actin. ^*^P<0.01 vs control (CTL); ^&^P<0.05 vs H/R; ^#^P<0.05 vs H/R or H/R + CAT (CAT); n = 3. (**B**) Bcl2 and Bax protein expression was detected by western blot. (**C**) Normalization of Bcl2 and Bax expression to α-Tubulin. ^*^P<0.01 vs control (CTL); ^&^P<0.05 vs H/R; ^#^P<0.05 vs. H/R or H/R + CAT (CAT); n = 4. (**D**) Bcl2/Bax ratio. ^*^P<0.01 vs control (CTL); ^&^P<0.05 vs H/R; ^#^P<0.05 vs H/R or H/R + CAT (CAT); n = 4.

To investigate the mechanism whereby PEP-1-CAT attenuates H/R-induced H9c2 apoptosis, we examined the expression of Bcl-2 and Bax. Both qRT-PCR and Western blot analyses showed that Bcl-2 expression was markedly increased in PEP-1-CAT-pretreated cells compared to the H/R or CAT-treated group. As expected, Bax expression was markedly decreased by PEP-1-CAT (Figure 4A-D), suggesting that PEP-1-CAT prevented H9c2 cells from apoptosis by increasing Bcl-2 while inhibiting Bax expression.

### PEP-1-CAT restored H/R-blocked mitochondrial membrane potential

**Figure 5:**
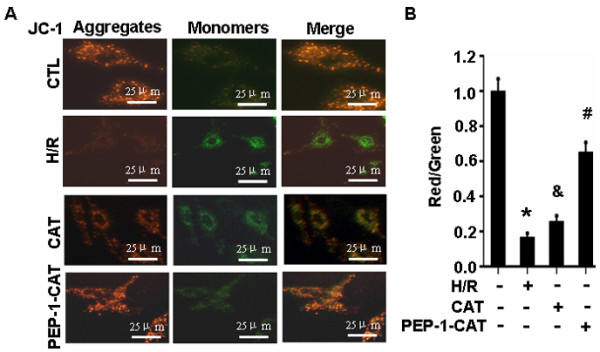
**PEP-1-CAT restored H/R-induced reduction of mitochondrial membrane potential.** (**A**) Mitochondrial transmembrane potential was assessed by the lipophilic cationic probe JC-1. Red signal indicates JC-1 aggregates in mitochondria. Green signal shows cytosolic JC-1 monomers indicative of the loss of mitochondrial membrane potential. (**B**) Quantitative analysis of membrane potential in (A). *P < 0.05 vs control (CTL); ^&^P < 0.05 vs H/R; ^#^P<0.05 vs H/R or H/R + CAT (CAT) group; n = 3.

Untreated cells exhibited bright-staining mitochondria that emitted red fluorescence. H/R treatment caused the formation of monomeric JC-1, indicative of loss of membrane potential. PEP-1-CAT pretreatment, however, blocked the HR-induced formation of JC-1 monomers (Figure 5A-B), suggesting PEP-1-CAT can restore H/R-induced loss of mitochondrial membrane potential.

### PEP-1-CAT inhibited H/R-induced H9c2 apoptosis through regulating multiple signaling pathways

**Figure 6:**
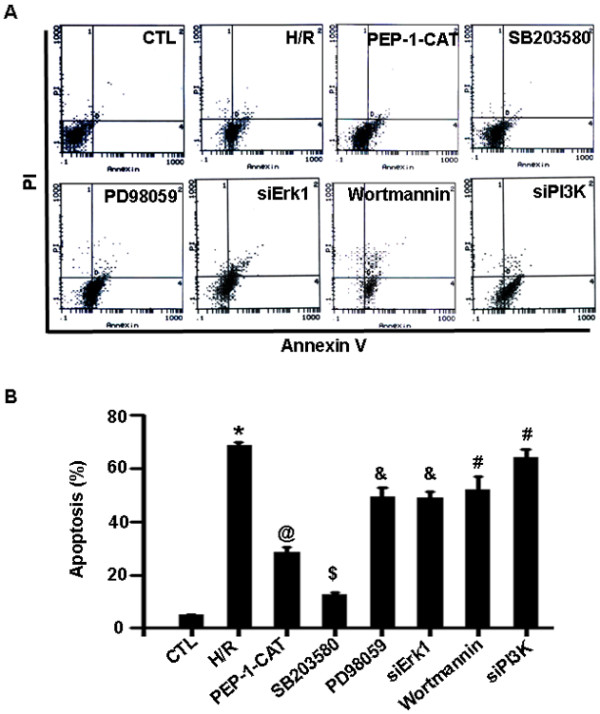
**PEP-1-CAT inhibited H/R-induced H9c2 apoptosis via p38, PI3K and Erk1/2 MAPK signaling pathways.** (**A**) H9c2 apoptosis was assessed by Flow Cytometry. (**B**) Quantification of H9c2 apoptosis rate. *P<0.01 vs CTL; ^@^P<0.01 vs H/R group; ^$^P<0.01 vs H/R or PEP-1-CAT-treated group; ^&^P<0.05 vs PEP-1-CAT-treated group; ^#^P<0.05 vs PEP-1-CAT-treated group; n = 5.

**Figure 7:**
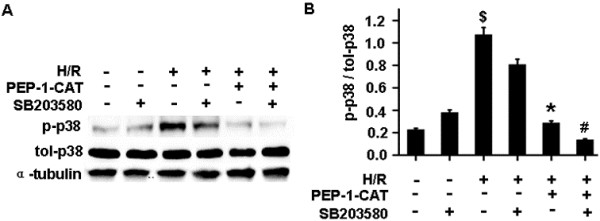
**PEP-1-CAT inhibited p38 phosphorylation.** (**A**) Western blot analysis showing PEP-1-CAT inhibited p38 phosphorylation. (**B**) Quantitative analysis of p38 phosphorylation by normalizing to total p38. ^$^P<0.01 vs CTL; ^*^P<0.05 vs H/R group; ^#^P<0.01 vs PEP -1-CAT-treated group; n = 4.

Previous studies have shown that apoptosis is mediated by multiple signaling pathways or protein factors including PI3K/Akt, p38 and Erk1/2 MAPK, etc. [13,14]. To determine which pathways are involved in PEP-1-CAT-mediated protection of H/R-injured H9c2 cells, we treated H9c2 with specific inhibitors for each individual pathways. We found that PI3K/Akt and Erk1/2 signaling pathways were essential for mediating PEP-1-CAT inhibition of H/R-induced apoptosis because PI3K/Akt inhibitor wortmannin, PI3K siRNA, Erk1/2 inhibitor PD98059, or Erk1 siRNA blocked PEP-1-CAT-induced reduction of H9c2 apoptosis (Figure 6A-B). p38 MAPK appeared to be also important for PEP-1-CAT function. Although p38 MAPK inhibitor did not reverse PEP-1-CAT-mediated decrease of H9c2 apoptosis (Figure 6A-B), PEP-1-CAT transduction inhibited p38 phosphorylation (Figure 7), suggesting that PEP-1-CAT blocks p38 signaling. These results demonstrated that PEP-1-CAT attenuated p38 signaling while enhancing PI3K and Erk1/2 MAPK signaling.

## Discussion

Myocardial apoptosis is a significant pathophysiological event in myocardial ischemia-reperfusion injury [9]. It is widely acknowledged that intervention of myocardial apoptosis is a very important approach to the prevention of myocardial ischemia-reperfusion injury [15]. Reperfusion causes myocardium to produce a large amount of ROS including superoxide anion (O_2_^-^·), hydroxyl radical (OH^-^), and hydrogen peroxide (H_2_O_2_), etc [16]. CAT, one of most important enzymes, can protect cells from oxidative damage. But its potential to be used to protect myocardium from H/R-induced injury is hindered by the poor permeability and the selectivity of cell membrane. By fusing CAT with a PEP-1 peptide, we were able to efficiently transduce PEP-1-CAT into H9c2 cells and protect myocardium from H/R-induced injury [10]. The present study advanced our previous finding by identifying novel mechanisms underlying PEP-1-CAT function in protecting cardiomyoctyes. We have found that PEP-1-CAT protects H/R-induced injury of H9c2 cells by restoring H/R-induced alteration of H9c2 morphology, inhibiting H/R-induced production of O_2_^-^·, and blocking LDH release and MDA production, the two indicators for hypoxia-reoxygenation injury [17,18].

ROS causes damages to intracellular macromolecules such as DNA breakage and lipid membrane peroxidation, leading to cell apoptosis [19]. Our data demonstrate that PEP-1-CAT blocks H/R-induced H9c2 apoptosis by regulating mitochondria-related apoptotic pathways. Recent studies have shown that H/R injury induces mitochondria to produce a high level of ROS [20,21]. Excessive ROS damages mitochondria, opens its permeability transition pore (PTP) and thus induces mitochondrial permeability transition (MPT), leading to mitochondrial depolarization and outer membrane rupture, which causes cell apoptosis or death [22,23]. Our studies indicate that H/R induces a decreased mitochondrial membrane potential, suggesting an impairment of mitochondria function. PEP-1-CAT transduction, however, restores mitochondrial membrane potential. These data demonstrate that PEP-1-CAT protects H9c2 cells from H/R-induced apoptosis by maintaining mitochondria membrane integrity and function of cardiomyocytes. Moreover, previous studies indicate that Bcl-2 family is upregulated during the opening of PTP [24]. Our results demonstrate that PEP-1-CAT regulates the expression of Bcl-2 family. PEP-1-CAT significantly increases Bcl-2 while decreasing Bax protein levels that are altered by H/R injury.

PEP-1-CAT prevents cardiomyocyte from H/R-induced injury by regulating multiple signaling pathways. Although a number of signaling pathways are involved in H/R-induced myocardial injury and apoptosis, PEP-1-CAT protects cardiomyocytes through down-regulation of p38 MAPK and activation of PI3K and Erk1/2 signaling pathways. PEP-1-CAT transduction inhibits p38 MAPK phosphorylation, suggesting that p38 MAPK mediates, at least in part, the function of PEP-1-CAT. Blockade of PI3K and Erk1/2 signaling significantly attenuates PEP-1-CAT-mediated reduction of H9c2 apoptosis, indicating that PI3K and Erk1/2 signaling pathways are essential for PEP-1-CAT activity in protecting cardiomocytes.

In summary, PEP-1-CAT transduction efficiently protects cardiomyocyte from H/R-induced apoptosis by blocking ROS production in mitochondria, which maintains mitochondria membrane integrity and inhibits the activation of Bcl2/Bax apoptotic pathway. Moreover, PEP-1-CAT blocks cardiomyocyte apoptosis by blocking p38 MAPK while activating PI3K and Erk1/2 MAPK signaling pathways. How these signaling pathways interact with each other in mediating PEP-1-CAT function will be a interesting subject for future study. Nevertheless, our study provides novel information and rationale for developing PEP-1-CAT as a therapeutic agent for treating or preventing myocardial ischemia-reperfusion injury.

## Competing interests

The authors declare that they have no competing interests.

## Authors’ contributions

LZ and SW designed and performed the experiments, collected the data and analyzed the results. JNW and JMT participated in the experimental design and interpretation of the results. LYG made fusion protein and evaluated the apoptosis by Flow Cytometry. FZ carried out Western blot. XK performed part of the in vitro experiments. JYY and YZH carried out the immunoassays. SYC assisted with writing the manuscript. All the authors have read and approved the final manuscript.

## Authors’ information

Co-first author: Lei Zhang and Shuang Wei.

## Supplementary Material

Additional file 1: Figure S1 Transduction of PEP-1-CAT into H9c2 cells. Click here for file
