# Supplementary material for: PEP-1-CAT protects hypoxia/reoxygenation-induced cardiomyocyte apoptosis through multiple sigaling pathways
Source: J Transl Med. 2013 May 6;11:113. doi: 10.1186/1479-5876-11-113 (PMC3660214; doi:10.1186/1479-5876-11-113)
Supplement: Additional file 1: Figure S1 — Transduction of PEP-1-CAT into H9c2 cells. [file 1479-5876-11-113-S1.doc]

**Additional file 1**

**PEP-1-CAT protects hypoxia/reoxygenation-induced cardiomyocyte apoptosis through multiple sigaling pathways**

Lei Zhang, Shuang Wei, Jun-Ming Tang, Ling-Yun Guo, Fei Zheng, Xia Kong, Jian-Ye Yang, Yong-Zhang Huang, Shi-You Chen, Jia-Ning Wang

**
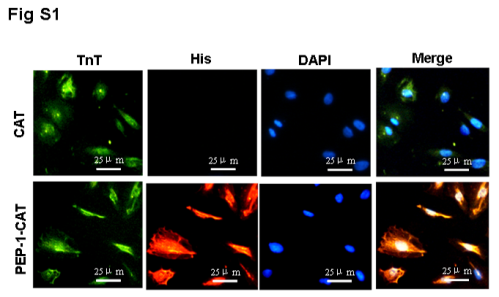
**

**Fig S1: Transduction of PEP-1-CAT into H9c2 cells.** H9c2 cells were treated with 2 μM purified CAT and PEP-1-CAT proteins for 6 h. The cells were incubated with FITC-conjugated mouse-anti Troponin T (TnT) and TRITC-conjugated rabbit-anti polyhistidine (His) antibodies and then visualized with fluorescent microscopy. Green signal shows TnT expression; Red signal indicates penetration of PEP-1-CAT; DAPI stains nuclei.
